# Mutational analyses of the interacting domains of *Schizosaccharomyces pombe* Byr2 with 14-3-3s

**DOI:** 10.1007/s00294-024-01293-7

**Published:** 2024-06-24

**Authors:** Yasuyo Kobayashi-Ooka, Fumiyo Ozoe, Makoto Kawamukai

**Affiliations:** 1 Department of Life Sciences, Faculty of Life and Environmental Sciences, Shimane University, 1060 Nishikawatsu, Matsue, 690-8504 Japan; 2 Nitto Denko Corporation Ibaraki Plant, 1-1-2, Shimohozumi, Ibaraki, Osaka 567-8680 Japan

**Keywords:** *S. pombe*, 14-3-3, MAP kinase, Byr2, Rad24, Rad25

## Abstract

The Byr2 kinase of fission yeast *Schizosaccharomyces pombe* is recruited to the membrane with the assistance of Ras1. Byr2 is also negatively regulated by 14-3-3 proteins encoded by *rad24* and *rad25*. We conducted domain and mutational analysis of Byr2 to determine which region is critical for its binding to 14-3-3 proteins. Rad24 and Rad25 bound to both the Ras interaction domain in the N-terminus and to the C-terminal catalytic domain of Byr2. When amino acid residues S87 and T94 of the Ras-interacting domain of Byr2 were mutated to alanine, Rad24 could no longer bind to Byr2. S402, S566, S650, and S654 mutations in the C-terminal domain of Byr2 also abolished its interaction with Rad24 and Rad25. More than three mutations in the C-terminal domain were required to abolish completely its interaction with 14-3-3 protein, suggesting that multiple residues are involved in this interaction. Expression of the N-terminal domain of Byr2 in wild-type cells lowered the mating ratio, because it likely blocked the interaction of Byr2 with Ste4 and Ras1, whereas expression of the catalytic domain of Byr2 increased the mating ratio as a result of freeing from intramolecular regulation by the N-terminal domain of Byr2. The S87A and T94A mutations of Byr2 increased the mating ratio and attenuated inhibition of Byr2 by Rad24; therefore, these two amino acids are critical for its regulation by Rad24. S566 of Byr2 is critical for activity of Byr2 but not for its interaction with 14-3-3 proteins. In this study, we show that 14-3-3 proteins interact with two separate domains in Byr2 as negative regulators.

**Supplementary Information:**

The online version contains supplementary material available at 10.1007/s00294-024-01293-7.

## Introduction

The fission yeast *Schizosaccharomyces pombe* proliferates continuously when it has abundant nutrients but arrests cell cycle progression in the G1 phase when it lacks nutrients, especially nitrogen. When opposite mating type cells are present, they begin conjugation and subsequently undergo meiosis, leading to the formation of four spores (Kawamukai 2024). The mating signal is recognized by the receptor named Map3 in *h*^+^ cells or Mam2 in *h*^−^cells, which couples with GTP binding protein Gpa1 to regulate Byr2 (Obara et al. 1991), a mitogen activating protein (MAP) kinase kinase kinase. Byr2 consists of a MAP kinase cascade with down-stream Byr1 MAP kinase kinase and Spk1 MAP kinase (Toda et al. 1991; Neiman et al. 1993; Yamamoto et al. 2004). Spk1 regulates Ste11, the key transcription factor for sexual development (Sugimoto et al. 1991; Kjaerulff et al. 2005), as well as the WD repeat protein Cpc2 and RNA-binding protein Msa2 (Paul et al. 2009; Oowatari et al. 2011).

Ras1 interacts with Byr2 (Masuda et al. 1995) to recruit it to the membrane to sense the mating signal from Gpa1 (Wang et al. 1991), which activates Byr2 (Bauman et al. 1998). The structure of the Ras-interacting domain spanning residues 71–180 of Byr2 has been solved (Gronwald et al. 2001). The structure of this domain consists of three alpha helices and a mixed five-stranded beta sheet arranged in the topology (Gronwald et al. 2001). Byr2 has a distinctive N-terminal kinase regulatory domain and a characteristic C-terminal kinase catalytic domain. Ste4 contains a SAM (sterile alpha motif domain) and a leucine zipper (Ste4-LZ) domain and acts as an adaptor protein of Byr2 (Barr et al. 1996). SAM domains are protein–protein interaction modules found in a large number of regulatory proteins. Ste4 interacts with the SAM domain of N-terminal Byr2 to form a Ste4–Byr2 complex (Tu et al. 1997). The regulatory domain and the catalytic domains of Byr2 interact, which results in autoinhibitory regulation.

Byr2 is maintained in an inactive form by binding to the 14-3-3 proteins Rad24 and Rad25. Overexpression of *rad24* or *rad25* reduces mating and sporulation in homothallic wild-type cells (Ozoe et al. 2002). By contrast, the mating and sporulation efficiencies of *rad24∆* or *rad25∆* cells are higher than those of wild-type cells, where *rad24∆* exhibits a much stronger phenotype than *rad25∆* cells (Zhou et al. 2000; Ozoe et al. 2002; Oowatari et al. 2009). A dominant negative form of the Rad24 mutant, Rad24-E185K, that fails to interact with Byr2, Mei2, and Ste11 results in a strong *sam* (skip starvation for mating) phenotype or hyper mating phenotype (Katayama et al. 1996; Gupta et al. 2011a, b; Ohshima et al. 2023).

In this study, to gain further insight into the interaction of Byr2 with 14-4-3 proteins, we explored the interacting domains and critical amino acid residues of Byr2 required for its binding to 14-3-3 proteins.

## Materials and methods

### Strains and media

The *S. pombe* strains used in this study are listed in Table 1. *S. pombe* cells were grown (Katayama et al. 1996) in YES-rich medium (0.5% yeast extract, 3% glucose, 225 mg/L adenine, histidine, leucine, uracil, and lysine hydrochloride) or minimum synthetic medium (EMM2) supplemented with 225 mg/L adenine, leucine, and/or uracil when necessary (Moreno et al. 1991).

**Table 1:** Strains used in this study

Strain	Genotype	Source
SP66	*h*^*90*^* ade6-M210 leu1-32*	Kawamukai (1999)
SP870	*h*^*90*^* ade6-M210 leu1-32 ura4-D1*8	Kawamukai (1999)
SP870A	*h*^*90*^* ade6-M216 leu1-32 ura4-D1*8	Kawamukai (1999)
SPSA	*h*^*90*^* ade6-M210 leu1-32 ura4-D18 byr2::ura4::ADE2*	Ozoe et al. (2002)

### Plasmids

Plasmids constructs are shown in Fig. S1. pSLF272 and pSLF273 were obtained from S. Forsburg (Forsburg and Sherman 1997). To clone regions of the *byr2* gene, the regions were amplified by PCR using appropriated forward and reverse primers (Table S1). They were first cloned into an intermediate vector such as pBluescript and then re-cloned into pSLF272 and pSLF273. To introduce mutations, site-directed mutagenesis was conducted using the Quik Change XL Site-directed mutagenesis kit (Stratagene). Mutations in the constructed plasmids were verified by DNA sequencing. Plasmids pREP41-Rad24-GFP and pREP41-Rad25-GFP were described previously (Ozoe et al. 2002).

### Immuno-precipitation and western blotting

*S. pombe* cells were grown in EMM2 with appropriate supplements to mid-logarithmic phase (~ total of 2 × 10^8^ cells), harvested by centrifugation, and washed once with ice-cold stop buffer (150 mM NaCl, 50 mM NaF, 10 mM EDTA, 1 mM NaN_3_ [pH 8]). The cells were lysed in 100 μL of ice-cold lysis buffer (50 mM Tris [pH 8.0], 150 mM NaCl, 0.8% Nonidet-P40, 5 μM EDTA, 10% glycerol, 1 mM phenylmethylsulfonyl fluoride, and protease inhibitor cocktail tablets [Complete; Boehringer Mannheim]) by vigorous vortexing with four 15-s pulses in the presence of 0.5-mm-diameter zirconia/silica beads (Biospec Products, Inc.). After centrifugation to sediment cells, the protein concentration in the supernatant was estimated by measuring absorbance at *A*_280_ and adjusted to 10 mg/mL with lysis buffer.

SPSA (*byr2∆*) cells expressing either GFP (control vector), Rad24-GFP, or Rad25-GFP were grown at 30 °C for 18 h in EMM2 lacking thiamine to induce *nmt41* promoter driven genes. Immunoprecipitation analysis was performed with anti-GFP antibody A-6455 (Molecular Probes) using a previously described method (Ozoe et al. 2002). Anti-HA monoclonal antibody sc-7392 (Santa Cruz Biotechnology) against HA was used for detection. Immunoprecipitates were suspended in sodium dodecyl sulfate loading buffer, immediately boiled for 5 min, and resolved by sodium dodecyl sulfate-10% polyacrylamide gel electrophoresis. Proteins were then transferred to a polyvinylidene difluoride membrane (Immobilon; Millipore). A horseradish peroxidase-conjugated goat anti-mouse IgG (Santa Cruz Biotechnology) was used as a secondary antibody to detect HA fusion proteins. The immunoreaction was visualized using the ECL kit (Amersham Pharmacia).

### Mating and sporulation efficiency assay

EMM2 medium and nitrogen-free EMM2 medium (1% glucose without ammonium chloride) was used measure the mating efficiency of *S. pombe*.

Mating and sporulation efficiencies were calculated using the following equation:

$\text{Mat }(\text{\%})\hspace{0.17em}=\hspace{0.17em}(2Z\hspace{0.17em}+\hspace{0.17em}2A\hspace{0.17em}+\hspace{0.17em}0.5S)/(H\hspace{0.17em}+\hspace{0.17em}2Z\hspace{0.17em}+\hspace{0.17em}2A\hspace{0.17em}+\hspace{0.17em}0.5S),$where *Z* stands for the number of zygotes, *A* for the number of asci, *S* for the number of free spores, and *H* for the number of cells that failed to mate. Mating ratio was calculated in duplicate samples.

## Results

### Rad24- and Rad25-interacting domains of Byr2

**Fig. 1:**
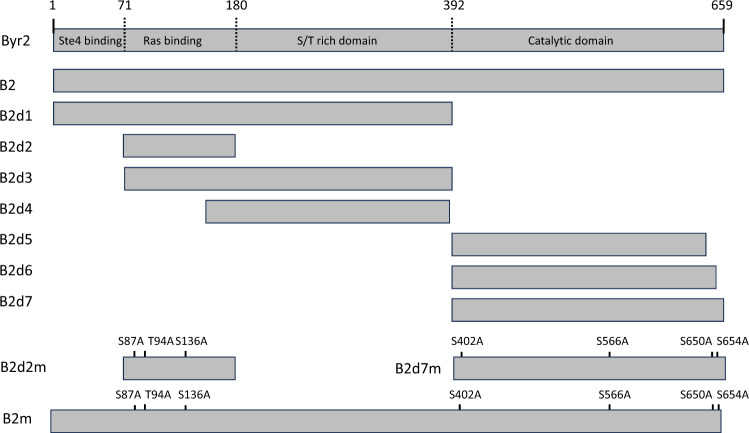
Domain structure of Byr2. Byr2 consists of four domains. The N-terminus region between residues 1–71 contains the Ste4 binding domain, the region between residues 71–180 contains the Ras-binding domain, the central region between residues 180–392 contains the S/T rich domain, and the C-terminus region between residues 392–659 contains the catalytic domain. Some regions of the *byr2* gene were cloned into pSLF272 or pSLF273 and expressed under the control of the *nmt41* promoter. B2m, B2d2m, and B2d7m regions containing Byr2 mutants in plasmid pSLF272 or pSLF273 are also shown

**Fig. 2:**
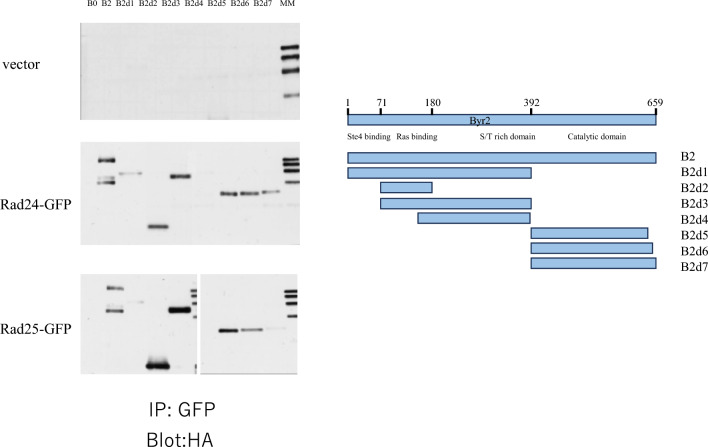
Rad24 and Rad25 interaction domains of Byr2. **A** SPSA strain (*h*^90^ *byr2::ura4::ADE2*) harboring plasmids pSLF272, pSLF272-B2, pSLF272-B2d1, pSLF272-B2d2, pSLF272-B2d3, pSLF272-B2d4, pSLF273-B2d5, pSLF273-B2d6, or pSLF273-B2d7 and pREP41-GFP, pREP41-rad24-GFP, or pREP41-rad25-GFP were grown on EMM2 medium to log phase. Anti-GFP antibody was used for immunoprecipitation of Rad24-GFP and Rad25-GFP and the immunoprecipitates were immunoblotted with anti-HA to detect Byr2-HA tagged proteins

It has been shown that Byr2 is negatively regulated by its interaction with 14-3-3 proteins encoded by *rad24* and *rad25* (Ozoe et al. 2002). To explore which regions of Byr2 interact with 14-3-3 proteins, we constructed plasmids expressing different regions of Byr2 tagged with HA. Byr2 was separated into seven regions (B2d1–B2d7) (Fig. 1) and each region was expressed in a SPSA (*byr2*∆) strain from the plasmid pSLF272 or pSLF273. Rad24 or Rad25 were also expressed as GFP fusions. For expression of B2d1–B2d4 and B2d5–B2d7, pSLF272 and pSLF273 vectors, respectively, were used (Fig. S1). Both vectors contain the same *nmt41* promoter, but the HA tag is located at the C-terminus of B2d1–B2d4, while in B2d5–B2d7, it is located in the N-terminus. We then examined their interaction by immunoprecipitation. Rad24 or Rad25 was immunoprecipitated with anti-GFP. Immunoblotting of the immunoprecipitates with anti-HA antibody detected various forms of Byr2. Six Byr2-derived proteins, B2d1, B2d2, B2d3, B2d5, B2d6, and B2d7, were co-precipitated with Rad24-GFP and Rad25-GFP, but B2d4 was not (Fig. 2). Interaction of B2d1 and B2d7 with Rad25-GFP was weak, but a band was still detected. Because B2d4 contains the S/T rich domain, but not the Ras1-interacting domain or C-terminal domain, the S/T rich domain is unlikely to be the domain that interacts with Rad24 and Rad25. Thus, the immunoprecipitation experiments indicated that the Ras-binding domain and the C-terminal domain interacted independently with 14-3-3s.

### Amino acid residues in the Ras-binding region and C-terminal region of Byr2 important for interaction with Rad24 and Rad25

**Fig. 3:**
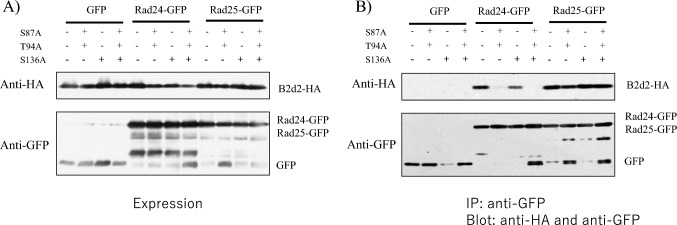
The Ras-interacting domain of the Byr2 mutant and its binding to Rad24 and Rad25. **A** The Ras-interacting domain mutants of Byr2 (B2d2, B2d2-S87A-T94A, B2d2-S136A, B2d-S87A-T94A-S136A) and Rad24-GFP or Rad25-GFP were expressed in the SPSA strain. Expression of B2d2 and B2d2 mutants was detected by anti-HA antibody, and Rad24-GFP and Rad25-GFP were detected by anti-GFP antibody. **B** The same strains were used for immunoprecipitation of Rad24-GFP and Rad25-GFP with ant-GFP and immunoprecipitates were immunoblotted with anti-HA antibody to detect Byr2-HA tagged proteins

The consensus sequence of 14-3-3 binding sites are reported to be RSXpSXP (Fu et al. 2000), but no sequence that completely matches this sequence is found in Byr2. Two S/TXP sequences, one situated at residue S136 in the Ras-binding domain and the other situated at residue S654 in the C-terminal domain, are found in Byr2 (Fig. S2). We also considered the 14-3-3 binding site in *S. pombe* Cdc25 (Zeng and Piwnica-Worms 1999) and selected seven amino acid residues to be mutated: three residues (S87, T94, and S136) in the Ras-binding region and four residues (S402, S566, S650, and S654) in the C-terminal region. Mutations were first introduced into the Ras-interacting domain of Byr2. S87A, T94A, and S136A mutations were introduced in the B2d construct expressing residues 71–180 of Byr2. We first verified that the expression level of various B2d mutants and Rad24-GFP or Rad25-GFP did not vary between the tested trains (Fig. 3A). Then, Rad24-GFP or Rad25-GFP was immunoprecipitated with anti-GFP antibody and the immunoprecipitates were blotted with anti-HA antibody to detect Byr2 containing residues 71–180 (Fig. 3B). Mutation of all three residues (S87A, T94A, and S136A) abolished the interaction with Rad24, while the S87A and T94A mutations compromised the interaction with Rad24. These mutations did not affect interaction with Rad25 (Fig. 3).

**Fig. 4:**
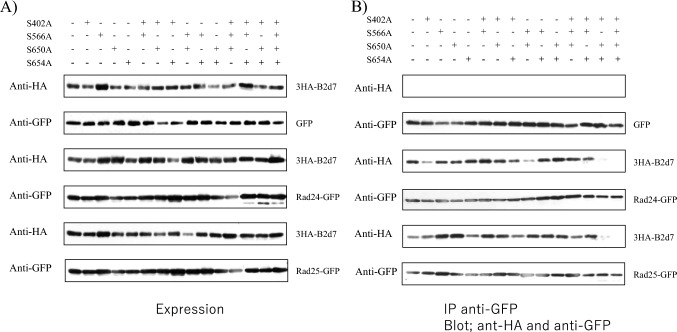
The C-terminal Byr2 mutants and their binding to Rad24 and Rad25. **A** The C-terminal domain mutants of Byr2 and Rad24-GFP or Rad25-GFP were expressed in the SPSA strain. Expression of B2d7 and B2d7 mutants was detected by anti-HA antibody, and Rad24-GFP and Rad25-GFP were detected by anti-GFP antibody. **B** The same strains were used for immunoprecipitation of Rad24-GFP and Rad25-GFP with ant-GFP antibody and immunoprecipitates were immunoblotted with anti-HA antibody to detect the C-terminal Byr2-HA tagged mutant proteins

We next introduced alanine substitutions at residues S402, S566, S650, and S654 in the C-terminal domain (residues 393–659) of the B2d7 construct of Byr2. The mutants were expressed from plasmid pSLF273 together with concomitant expression of Rad24-GFP or Rad25-GFP in the SPSA strain (Fig. 4). We first verified that the expression levels of various B2d7 mutants and Rad24-GFP or Rad25-GFP did not vary between the tested trains (Fig. 4A). Rad24 or Rad25 was immunoprecipitated with anti-GFP antibody and the immunoprecipitates were blotted with anti-HA antibody to detect the Byr2 region including the 393–659 residues (Fig. 4B). The mutation of four amino acid residues (S402, S566, S650, and S654) to alanine abolished the interaction of the B2d7 construct with Rad24 and Rad25, and the mutation of three amino acid residues (S402, S650, and S654) to alanine compromised the interaction of the B2d7 construct of Byr2 with Rad24 and Rad25.

### Mutation of full-length Byr2

**Fig. 5:**
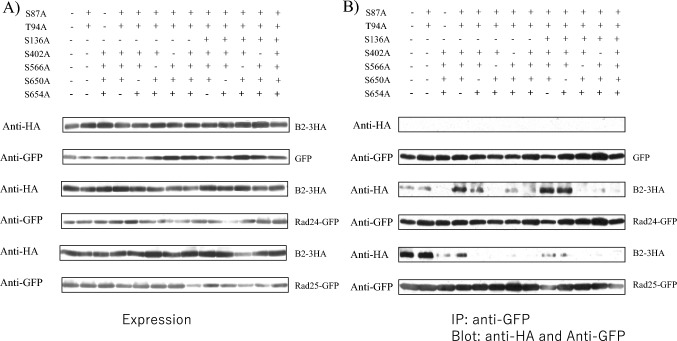
Interaction of Rad24 and Rad25 with point mutants of full-length *byr2*. **A** Mutants of full-length Byr2 and Rad24-GFP or Rad25-GFP were expressed in the SPSA strain. Expression of Byr2 and Byr2 mutants was detected by anti-HA antibody, and Rad24-GFP and Rad25-GFP were detected by anti-GFP antibody. **B** The same strains were used for immunoprecipitation of Rad24-GFP and Rad25-GFP with anti-GFP antibody and immunoprecipitates were immunoblotted with anti-HA antibody to detect full-length Byr2-HA tagged mutant proteins

To see how the mutations in the full-length Byr2 affected its interaction with 14-3-3s, Byr2 mutants were expressed from the plasmid pSLF272 together with concomitant expression of Rad24-GFP or Rad25-GFP. We first verified that the expression levels of various Byr2 mutants and Rad24-GFP or Rad25-GFP did not vary between the tested strains (Fig. 5A). Rad24-GFP or Rad25-GFP was immunoprecipitated with anti-GFP antibody and the immunoprecipitates were blotted with anti-HA to detect full-length Byr2 (Fig. 5B). Mutation of all seven residues (S87A, T94A, S136A, S402A S566A, S650A, and S654A) abolished the interaction of Byr2 with Rad24 and Rad25. Introduction of five mutations (S87A, T94A, S402A S650A, and S654A) into full-length Byr2 abolished the interaction of Byr2 with Rad24 and Rad25. Mutations of additional residues including these five mutations failed to interact with Rad24 or Rad25. The S402A, S566A, S650A, and S654A mutants failed to interact with Rad24 but weakly interacted with Rad25. The remaining constructs retained their interactions with Rad24 and Rad25. To disable the interaction of Byr2 with 14-3-3s, mutations of amino acids in the Ras-binding domain as well as in the C-terminal domain were necessary, supporting the observation that 14-3-3s interact with Byr2 via both regions.

### Expression of the Byr2 domain and mutants affects mating

As 14-3-3 proteins interacted separately with the N- and C-terminal domains of Byr2 (Fig. 1), we next tested the effects of expression of different domains of Byr2 on mating. The mating ratios of wild-type strains harboring different plasmids were examined by incubating them on EMM2-N plates at 30 °C for 7 h (Table 2). Expression of B2d8 and B2d9 constructs bearing the N-terminal domain of Byr2 lowered the mating ratio to 10.54% and 14.26%, respectively, while the mating ratio of the wild-type strain (SP870A) harboring the empty vector was 29.6% (Table 2). Expression of the Ras-interacting domains B2d1 and B2d2 lowered the ratio to 17.05% and 15.39%, respectively. Expression of the C-terminal domain (B2d7) of Byr2 increased the mating ratio to 39.88% which is consistent with previous observations (Kjaerulff et al. 2005).

**Table 2:** Mating ratio in WT by expression of a variety of truncated Byr2

	EMM-N	Relative %
vector	29.6 ± 7.8	100
Byr2	35.09 ± 12.31	118.5
B2d1	17.05 ± 4.95	57.6
B2d2	15.39 ± 5.31	52.0
B2d3	28.52 ± 7.28	96.4
B2d4	26.62 ± 8.88	89.0
B2d7	39.88 ± 4.62	134.7
B2d8	10.54 ± 7.66	35.6
B2d9	14.26 ± 11.5	48.2

**Fig. 6:**
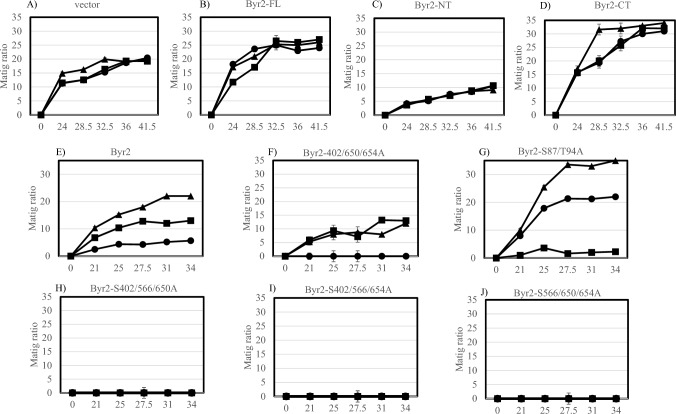
Effect of expression of Byr2 mutants on mating efficiency in cells expressing Rad24 or Rad25. The SP870A strain harboring pSLF172 (**A**), pSLF172-B2 expressing full-length Byr2 (**B**), pSLF172-B2d1 expressing the N-terminal region of Byr2 (**C**), or pSLF172-B2d7 expressing the C-terminal region of Byr2 (**D**) together with pREP41 (▲), pREP41-rad24 (●) or pREP41-rad25 (■) was used to examine mating ratios. The SPSA (*byr2∆*) strain harboring plasmids pSLF172-B2 (E), pSLF172-B2-S402A-S650A-S654A (F), pSLF172-B2-S87A-T94A (G), pSLF172-B2-S402A-S566A-S650A (H), pSLF172-B2-S402A-S566A-S654A (I), or pSLF172-B2-S566A-S650A-654A (J) together withpREP41 (▲), pREP41-rad24 (●) or pREP41-rad25 (■) was used to examine mating ratios. Cells were grown on EMM2 plates for indicated times (h) and 1,000 cells were counted for mating and sporulation. Mean and standard deviation of duplicate samples are shown

Expression of Rad24 or Rad25 in the wild-type strain lowers the mating ratio, as reported previously (Ozoe et al. 2002). We examined how the expression of N-terminal Byr2, C-terminal Byr2, or full-length Byr2 in cells co-expressing Rad24 or Rad25 affected mating ratios after incubation for 24–41.5 h on EMM2 plates (Fig. 6). In these experiments, nitrogen-containing medium was used. While the mating ratio in the strain harboring the vector control was 15–20% (Fig. 6A), expression of full-length Byr2 elevated the mating ratio to 25–30% and expression of Rad24 slowed the time to maximum mating (Fig. 6B). The mating ratio in the strain expressing the N-terminal domain of Byr2 was 5–10% (Fig. 6C) after the same period of incubation. Expression of Rad24 or Rad25 did not affect the mating ratio in the strain expressing the N-terminal domain of Byr2 (Fig. 6C), indicating a dominant negative effect of the N-terminal domain of Byr2 on mating is not interrupted by expression of 14-3-3 proteins. Expression of the C-terminal domain of Byr2 rapidly increased the mating ratio to 30–35% (Fig. 6D) and expression of Rad24 or Rad25 reduced the time needed to observe the increase in the mating ratio. These results clearly show a negative regulation of the C-terminal function of Byr2 by 14-3-3 proteins.

Because we observed some mutations in Byr2 compromised their interaction with 14-3-3 proteins (Fig. 5), we next monitored the mating efficiency of the SPSA (*byr2∆*) strain harboring different plasmids expressing mutant types of Byr2. While introduction of some mutations into Byr2 completely abolished its function, S402A, S650A, S654A or S87A and T97A mutations did not affect Byr2 function (Fig. 6F, G). The inhibitory effect of Rad25 was clearly reduced in the strain expressing the Byr2–S402A–S650A–S654A mutant, but mating was still inhibited by Rad24 expression (Fig. 6F). Introduction of the mutations S402A, S566A, and S650A, the mutations S566A, S650A, and S654A, or the mutations S402A, S566A, and S654A into full-length Byr2 abolished its complementary function in the *byr2∆* strain (Fig. 6H, I, J). Therefore, S566 is crucial for Byr2 function. It was difficult to test the effect of 14-3-3s in the constructs containing the S566A mutation. Introduction of S87A and S94A into full-length Byr2 rather elevated Byr2 activity in the *byr2* deletion strain. In this stain, the inhibitory effect of Rad24 was moderate, unlike Rad25, which still inhibited mating (Fig. 6G), supporting the observation that the Byr2–S87A–S94A mutant was still able to interact with Rad25 (Fig. 3). Because Rad24 and Rad25 interact with other proteins such as Ste11 and Mei2 (Kitamura et al. 2001), the effect of 14-3-3s on mating needs to take into consideration of these proteins’ role in addition to Byr2.

## Discussion

In this study, we explored the domains and amino acid residues of Byr2 that are important for its interaction with 14-3-3s encoded by *rad24* and *rad25* in the fission yeast *S. pombe*. Byr2 interacted via two sites with 14-3-3s, the Ras-binding domain in the N-terminal region and the C-terminal catalytic domain. Mutation of S87 and T94 to alanine abolished the binding of the Ras-interacting domain of Byr2 to Rad24; therefore, these amino acids are important for Byr2 interaction with Rad24. The expression of the Byr2–S87A–T94A mutant increased the mating ratio relative to wild-type Byr2. The inhibitory effect of Rad24 on the Byr2–S87A–T94A mutant was reduced; therefore, negative regulation of Byr2 by Rad24 was partly compromised by these mutations. The result confirms that residues S87 and T94 are important for negative regulation of Rad24 (but not so much of Rad25 under our experimental conditions) binding to the N-terminal domain of Byr2. We suggest that the interaction of 14-3-3 with this region of Byr2 is a regulatory mechanism that prevents the interaction of Ras1 with Byr2.

The interaction of Rad24 and Rad25 with the C-terminal domain region of Byr2 was abolished by the Byr2 mutations S402A, S566A, S650A, and S654A, while the interaction was not abolished when the same mutations were introduced into full-length Byr2. This indicates that the S402A, S566A, S650A, and S654A mutations in the N-terminal region of Byr2 do not negatively affect Byr2 interaction with 14-3-3s. Similarly, the full-length Byr2–S87A–T94A mutant retained its ability to interact with 14–3-3s. These results support the observation that 14–3-3s interact independently with the N-terminal and C-terminal domains of Byr2. When mutations were introduced at residues S402, S650, and S654 of Byr2, which abolished the interaction of 14–3-3s with the C-terminal region of Byr2 and this *byr2*-S402A, S650A, and S654A triple mutant was expressed in *byr2∆* strain (Fig. 6), the function of Byr2 was reduced and negative regulation by Rad25 was lost as indicated by the change in the mating ratio. These mutations in the catalytic domain affected the function of Byr2, but at the same time the regulation of Byr2 by Rad25 was lost. We consider that binding of Rad25 to the C-terminal domain of Byr2 has a negative regulatory role. We also observed that when the S566A mutation was included, the complementation of *byr2* mutants in the *byr2∆* strain was lost, indicating that S566 is crucial for Byr2 function, which is consistent with a previous report showing that S566A or S566E hardly rescue sporulation in *ras1*∆ cells (Bauman and Albright 1998). Since S566 was not a critical residue for Byr2 interaction with 14-3-3s, the regulatory role of this residue is still unclear.

For the analysis of the mating ratio, it is necessary to consider that Rad24 and Rad25 also bind to Ste11 and Mei2, particularly in their phosphorylated forms (Kitamura et al. 2001; Sato et al. 2002; Ohshima et al. 2023). These regulators play a central role in controlling sexual development, since the key transcription factor Ste11 and the RNA binding protein Mei2 promote the meiotic cycle. Although regulation of Byr2 was free from Rad24 and Rad25 by mutations in Byr2, these 14-3-3 proteins also affect the function of Ste11 and Mei2. Therefore, to assess the effect of Rad24 and Rad25 on sexual development, the regulation of these two other proteins needs to be considered. Nevertheless, we found that the S87A and T94A mutants of Byr2, which could not interact with Rad24, increased the mating ratio.

Our observation indicates that the binding affinities of Rad 24 and Rad25 for Byr2 are not identical. Rad25 expression had a clearer negative effect than that of Rad24 in the strain expressing the Byr2–S87A–T94A mutant. This is consistent with the result showing that Rad25 still interacts with the Byr2–S87A–T94A mutant. Because Rad24 and Rad25 proteins were highly expressed under the *nmt41* promoter without thiamine under our experimental conditions, the amounts of these proteins in this strain were probably equivalent. However, this may not reflect the physiological situation, because Rad24 is sixfold more abundant than Rad25 in wild-type cells (Ozoe et al. 2002). Although we observed a difference in the affinity of Rad24 and Rad25 for Byr2 and effect on the mating ratio in our experimental conditions, we think that the results need to be interpreted carefully considering the physiological context.

Byr2 is thought to be an ortholog of mammalian Raf1 kinase, because both kinases share the similar regulation by Ras proteins to receive extracellular signals in the plasma membrane. Ras-mediated Raf-1 recruitment to the plasma membrane maintains signaling fidelity. 14-3-3 interacts with the N-terminus regulatory domain (256–261) and the C-terminus kinase domain (618–623) of Raf1 (Light et al. 2002). Interaction of the N-terminus regulatory domain of Raf1 with 14-3-3 results in negative regulation, while the role of the interaction with the C-terminal domain remains undecided. The interaction of Rad24 with Raf1 was demonstrated by expressing Raf1 in *S. pombe* (Lee and Yoo 2007), indicating high conservation of 14–3-3 function between mammals and *S. pombe*. We showed previously that Ras1 recruits Byr2 to the membrane and 14-3-3 has a negative effect on this recruitment (Ozoe et al. 2002). Deletion of *rad24* or *rad25* increases sporulation efficiency in *ras1∆* diploid cells but not in *byr2∆* cells. Rad24 and Rad25 have no effect on the activity of constitutively active Byr1(S214DT218D) (Ozoe et al. 2002; Yamamoto et al. 2004), which supports the negative effect of 14-3-3 on Byr2.

**Fig. 7:**
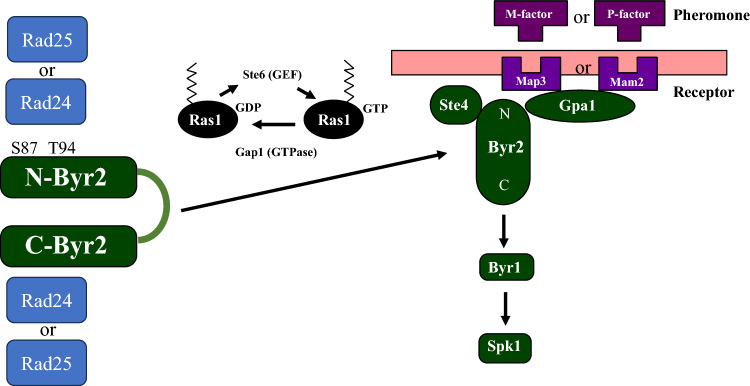
A model of the regulation of Byr2 by Rad24 and Rad25. Under nutrient-rich conditions, Byr2 is maintained in its inactive form by binding to Rad24 or Rad25. In this situation, Byr2 might maintain a closed conformation via the interaction between its N-terminal regulatory region and its C-terminal kinase domain. Under nutrient-poor conditions, *ste6,* encoding the GTP-GDP exchange factor of Ras1, is expressed, resulting in the activation of Ras. Activated Ras1 competes out 14-3-3 from Byr2 to recruit Byr2 to the membrane to receive pheromone signals from Gpa1. For efficient signaling, Ste4 bridges the formation of the Byr2 complex

Finally, we propose a model for the regulation of Byr2 based on the current data and previous results (Fig. 7). Under nutrient-abundant conditions, Byr2 remains inactive by binding to Rad24 or Rad25. In this situation, Byr2 might be kept in a closed conformation by the interaction between its N-terminal regulatory region and its C-terminal kinase domain (Tu et al. 1997) with the assistance of 14-3-3 proteins. Under nutrient-poor conditions, *ste6,* encoding the GTP-GDP exchange factor of Ras1, is expressed, resulting in the activation of Ras (Hughes et al. 1994). Activated Ras1 competes out 14-3-3 from Byr2 to recruit Byr2 to the membrane to receive pheromone signaling from Gpa1. For efficient signaling, Ste4 bridges the formation of the Byr2 complex (Ramachander et al. 2002).

## Supplementary Information

Below is the link to the electronic supplementary material.

Supplementary file1 Figure S1. Plasmid construct used in this study. Regions of the byr2 gene were amplified by PCR and cloned into the indicated sites of pSLF272 or pSLF273. The plasmids containing the byr2 gene regions are described in this figure. pSLF272 and pSLF273 contain the nmt41 promoter and 3HA tagging Byr2 either at the C-terminus or N-terminus. Figure S2. Amino acid sequence of Byr2 and seven mutation sites. Seven (S87, T94, S136, S402, S566, S650, and S654) amino acids residues in Byr2 were substituted with alanine in this study (PPTX 59 KB)

Supplementary file2 (DOCX 25 KB)

## Data Availability

All data generated or analysed during this study are included in this published article and its supplementary information files.
