# Supplementary material for: Mutational analyses of the interacting domains of Schizosaccharomyces pombe Byr2 with 14-3-3s
Source: Curr Genet. 2024 Jun 24;70(1):8. doi: 10.1007/s00294-024-01293-7 (PMC11196315; doi:10.1007/s00294-024-01293-7)
Supplement: Supplementary file 1 — Supplementary file1 Figure S1. Plasmid construct used in this study. Regions of the byr2 gene were amplified by PCR and cloned into the indicated sites of pSLF272 or pSLF273. The plasmids containing the byr2 gene regions are described in this figure. pSLF272 and pSLF273 contain the nmt41 promoter and 3HA tagging Byr2 either at the C-terminus or N-terminus. Figure S2. Amino acid sequence of Byr2 and seven mutation sites. Seven (S87, T94, S136, S402, S566, S650, and S654) amino acids residues in Byr2 were substituted with alanine in this study (PPTX 59 KB) [file 294_2024_1293_MOESM1_ESM.pptx]

## Slide 1
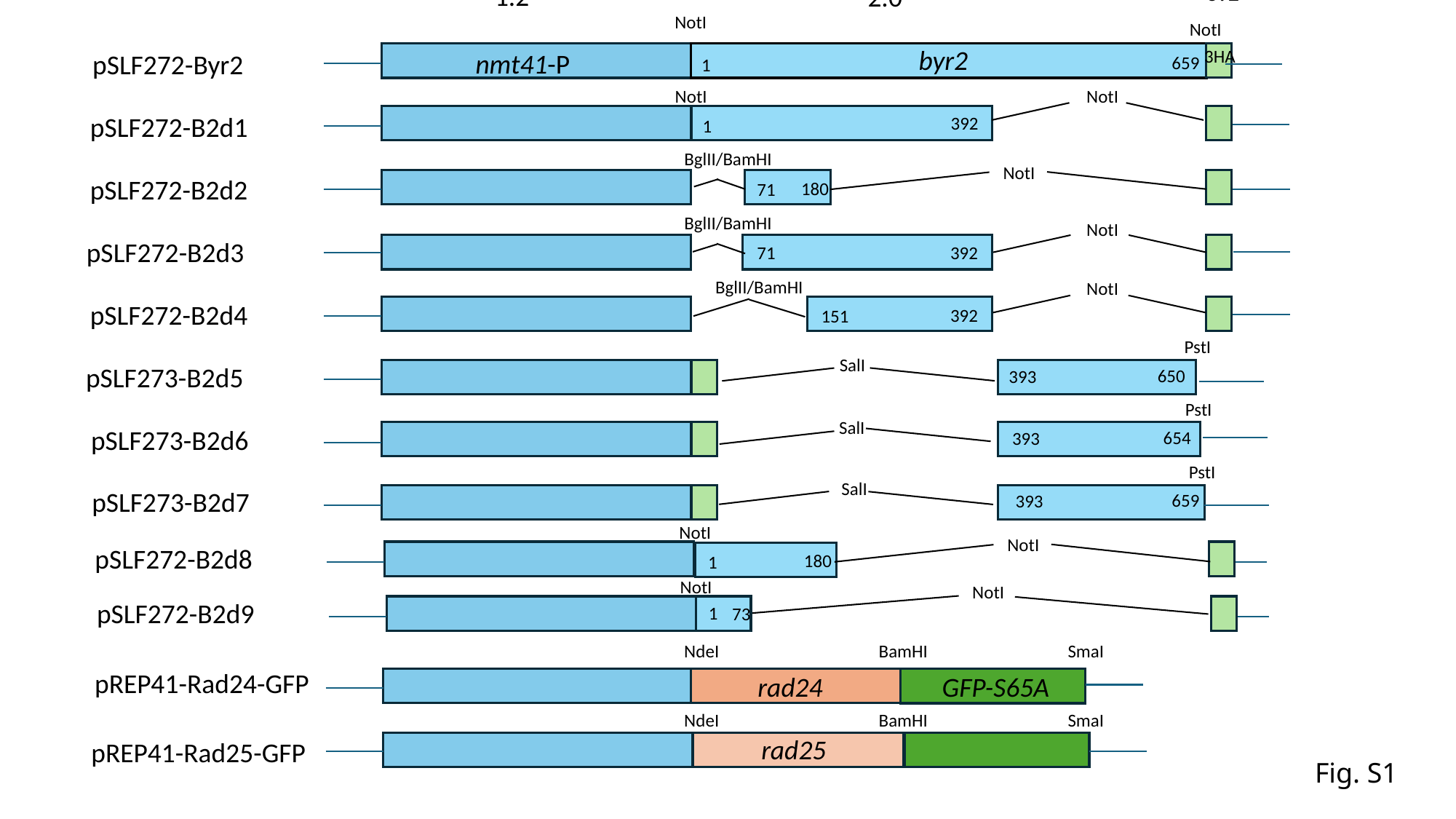

0.1
1.2
2.0
NotI
NotI
byr2
3HA
nmt41-P
pSLF272-Byr2
659
1
NotI
NotI
pSLF272-B2d1
392
1
BglII/BamHI
NotI
pSLF272-B2d2
180
71
BglII/BamHI
NotI
pSLF272-B2d3
392
71
BglII/BamHI
NotI
pSLF272-B2d4
392
151
PstI
SalI
pSLF273-B2d5
650
393
PstI
SalI
pSLF273-B2d6
654
393
PstI
SalI
pSLF273-B2d7
659
393
NotI
NotI
pSLF272-B2d8
180
1
NotI
NotI
pSLF272-B2d9
1
73
NdeI
BamHI
SmaI
pREP41-Rad24-GFP
GFP-S65A
rad24
NdeI
BamHI
SmaI
rad25
pREP41-Rad25-GFP
Fig. S1

## Slide 2
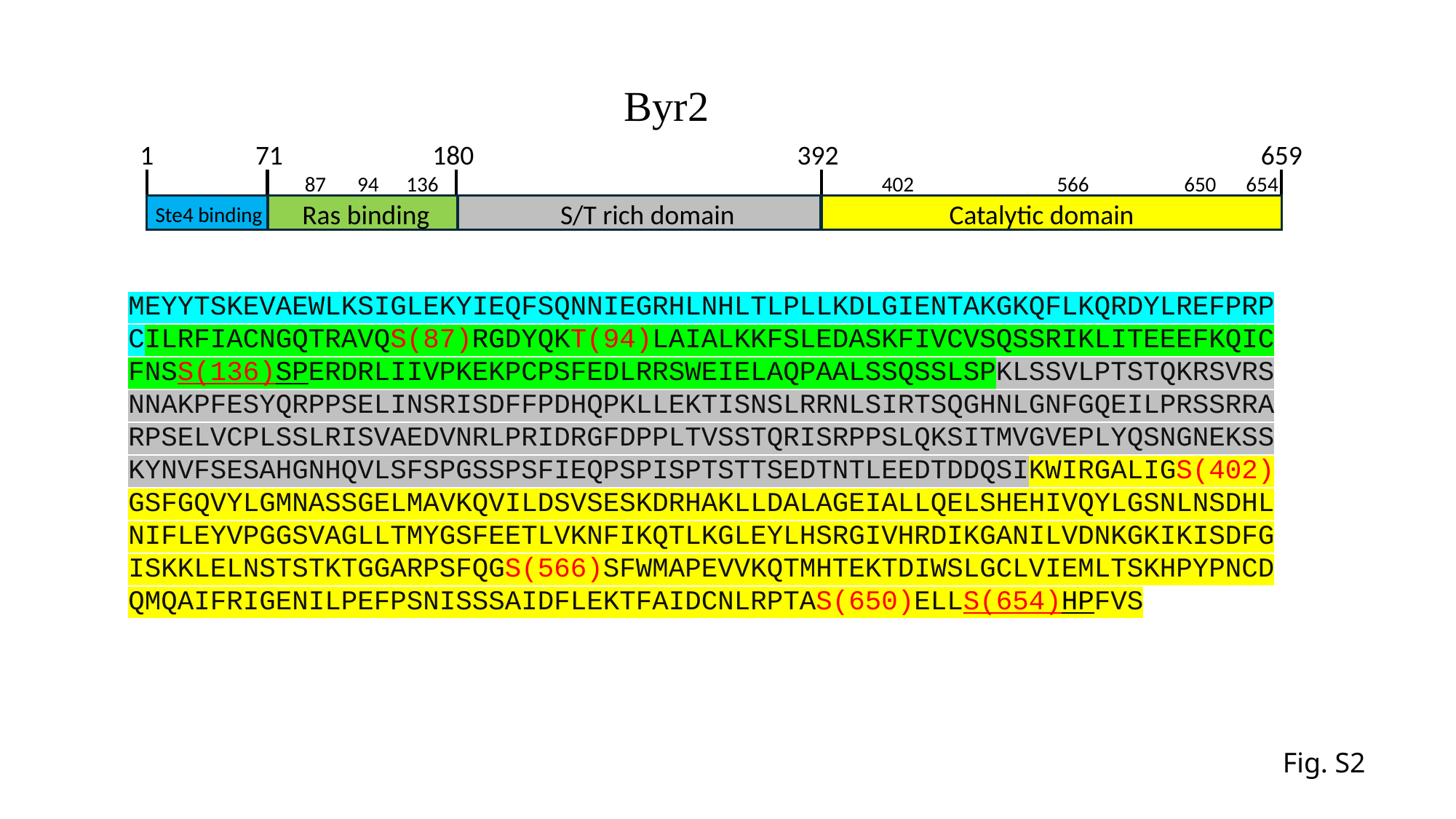

Byr2
1
71
180
392
659
87
94
136
402
566
650
654
Ras binding
S/T rich domain
Catalytic domain
Byr2
Ste4 binding
MEYYTSKEVAEWLKSIGLEKYIEQFSQNNIEGRHLNHLTLPLLKDLGIENTAKGKQFLKQRDYLREFPRPCILRFIACNGQTRAVQS(87)RGDYQKT(94)LAIALKKFSLEDASKFIVCVSQSSRIKLITEEEFKQICFNSS(136)SPERDRLIIVPKEKPCPSFEDLRRSWEIELAQPAALSSQSSLSPKLSSVLPTSTQKRSVRSNNAKPFESYQRPPSELINSRISDFFPDHQPKLLEKTISNSLRRNLSIRTSQGHNLGNFGQEILPRSSRRARPSELVCPLSSLRISVAEDVNRLPRIDRGFDPPLTVSSTQRISRPPSLQKSITMVGVEPLYQSNGNEKSSKYNVFSESAHGNHQVLSFSPGSSPSFIEQPSPISPTSTTSEDTNTLEEDTDDQSIKWIRGALIGS(402)GSFGQVYLGMNASSGELMAVKQVILDSVSESKDRHAKLLDALAGEIALLQELSHEHIVQYLGSNLNSDHLNIFLEYVPGGSVAGLLTMYGSFEETLVKNFIKQTLKGLEYLHSRGIVHRDIKGANILVDNKGKIKISDFGISKKLELNSTSTKTGGARPSFQGS(566)SFWMAPEVVKQTMHTEKTDIWSLGCLVIEMLTSKHPYPNCDQMQAIFRIGENILPEFPSNISSSAIDFLEKTFAIDCNLRPTAS(650)ELLS(654)HPFVS
Fig. S2
