# Supplementary material for: Mutational analyses of the interacting domains of Schizosaccharomyces pombe Byr2 with 14-3-3s
Source: Curr Genet. 2024 Jun 24;70(1):8. doi: 10.1007/s00294-024-01293-7 (PMC11196315; doi:10.1007/s00294-024-01293-7)
Supplement: Supplementary file 2 — Supplementary file2 (DOCX 25 KB) [file 294_2024_1293_MOESM2_ESM.docx]

**Table S1 Primers used in this study**

| Name | Sequence (5’→3’) |
| --- | --- |
| Byr2-1-F-Not | TAGCGGCCGCAATGGAATATTATACCTCG |
| Byr2-71-F-Bam | AAGGATCCCATGTGTATACTGAGGTTTA |
| Byr2-151-F-Bam | AAGGATCCAATGCCTTGTCCATCGTTTG |
| Byr2-393-F-Sal | TTGTCGACCAAATGGATACGTGGTG |
| Byr2-180-R-Not | TTGCGGCCGCTAGGAGAAAGGGAGGAC |
| Byr2-392-R-Not | ATGCGGCCGCTGATAGACTGATCATC |
| Byr2-645-R-Pst | AACTGCAGTTAAAGATTACAATCTATG |
| Byr2-650-R-Pst | AACTGCAGTTATGATGCTGTAGGTCTA |
| Byr2-659-R-Pst | TTCTGCAGTTATGAAACGAACGGGTGGGCCAACAACTC |
| Byr2-659-R-Not | TTGCGGCCGCATGAAACGAACGGGTG |
| B2m-82Xho-S87A-T94A-F | AACTCGAGCAGTTCAAGCACGAGGAGATTACCAGAAAGCACTC |
| B2m-S136A-F | CAAATTTGTTTTAATTCAGCTTCACCGG |
| B2m-S402A-F | GGTGCGTTAATAGGTGCAGGATCGTTTGG |
| B2m-S566A-F | CCCTTCTTTTCAAGGCGCTTCTTTTTGG |
| B2m-S650A-F | TAATCTTAGACCTACAGCAGCAGAGTTGT |
| B2m-S136A-R | CCGGTGAAGCTGAATTAAAACAAATTTG |
| B2m-S402A-R | CCAAACGATCCTGCACCTATTAACGCACC |
| B2m-S566A-R | CCAAAAAGAAGCGCCTTGAAAAGAAGGG |
| B2m-S650A-R | ACAACTCTGCTGCTGTAGGTCTAAGATTA |
| B2m-S654A-R-Not | TAGCGGCCGCATGAAACGAACGGGTGGGCCAACAACTC |

Double underlined ATG is a practical initiation codon to express protein.

Underlined codons indicate the mutation site.
